# General Practitioners’ Experiences of Clinical Consultations With Refugees Suffering From Mental Health Problems

**DOI:** 10.3389/fpsyg.2020.00412

**Published:** 2020-03-13

**Authors:** Samantha Marie Harris, Per-Einar Binder, Gro Mjeldheim Sandal

**Affiliations:** ^1^Department of Psychosocial Science, Faculty of Psychology, University of Bergen, Bergen, Norway; ^2^Department of Clinical Psychology, Faculty of Psychology, University of Bergen, Bergen, Norway

**Keywords:** general practitioner, refugee, mental health, barriers, challenges, facilitators, qualitative, interviews

## Abstract

Refugees suffer from higher rates of certain mental health problems than non-refugee migrants and the native population of their host country. General practitioners (GPs) in Norway and many other European countries are the first contact person for settled refugees in need of non-emergency medical support. This includes psychiatric support, although GPs are not typically specialists in psychiatry. The aim of this study is to investigate how GPs experience working with refugees suffering from mental health problems, with a specific focus on perceived challenges and facilitators. We conducted semi-structured interviews with 15 GPs working in Norway (7 females). Participants ages ranged from 29 to 67 (*M* = 41.7 years, *SD* = 11.1) with work experience ranging from 2 to 39 years (*M* = 13.6 years, *SD* = 12.1). Interviews were analysed thematically using the qualitative data analysis computer software package NVivo 12. The main challenges presented by GPs relate to language barriers, mismatched expectations, different understandings of health and illness, and the GP feeling unprepared to work with this patient group. The main facilitating themes related to establishing trust and finding the work meaningful. The themes presented in this study highlight areas of interest for future research, and should inform training programmes to improve health care for both clinicians and refugee patients.

## Introduction

General practitioners (GPs) experience a range of challenges that contribute to the perceived complexity of clinical consultations with refugees suffering from mental health problems. There are almost 26 million refugees on the planet today (UNHCR, 2019), who flee their home countries ‘because of persecution, war or violence’ and ‘have a well-founded fear of persecution for reasons of race, religion, nationality, political opinion or membership in a particular social group’ (UNHCR, 2017). According to Statistics Norway (2019), people with a refugee background currently constitute 4.4% (over 230,000 people) of Norway’s population. Studies have suggested that refugees experience higher rates of certain mental health problems such as anxiety, depression (Lindert et al., 2009), and post-traumatic stress disorder (PTSD) than non-refugee migrants or the native population of their host country (Fazel et al., 2005). This may also be the case for schizophrenia and other non-affective psychoses (Hollander et al., 2016). Due to experiences pre, during, and post flight they are faced with a range of mental and physical health problems (Matlin et al., 2018; World Health Organization [WHO], 2018), which are a challenge for host countries to meet adequately (Matlin et al., 2018). In many European countries, including the country of our study, Norway, the GP acts as a gatekeeper to specialist health services. While GPs are not typically specialists in psychiatry, they are required to make important decisions regarding patients’ mental health treatment. Clinicians have reported feeling they lack the resources or training to meet the demands placed on them by working with refugees suffering from mental health problems (Jensen et al., 2013; Wylie et al., 2018). This may be partly related to practical challenges such as language barriers, working with interpreters, patients’ illiteracy, and time constraints (Robertshaw et al., 2017). However, implicit challenges such as different understandings of illness and expectations of treatment (Jensen et al., 2013; Wylie et al., 2018), patients’ high expectations of health care professionals, and different cultural values (Robertshaw et al., 2017) may also play a role. Health care professionals working with patients with a different cultural background have previously reported feelings of distress, overload, and exhaustion as a result of this work (Terraza-Nunez et al., 2011).

**FIGURE 1:**
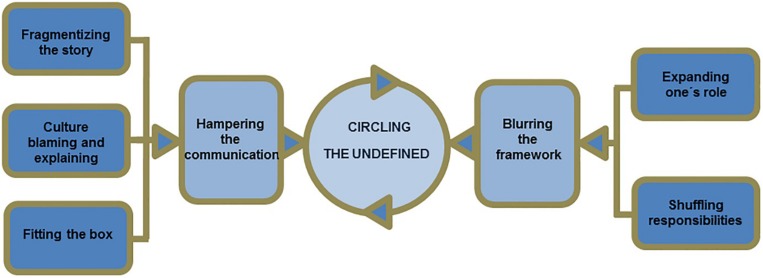
Rothlind et al. (2018) conceptual model describing how clinicians and patients continue to ‘circle the undefined’ through their behaviours in intercultural consultations.

Rothlind et al. (2018) developed a conceptual model of communication in intercultural primary care consultations, which gives an insight into the possible mechanisms underlying the perceived complexity of intercultural consultations. Their model is based on interviews with both patients with an immigrant background and their physicians, called ‘circling the undefined’ (Figure 1). They postulate that patients and their clinicians lack a shared understanding of issues that are fundamental to the consultation, such as what constitutes health and illness in the first place. They present themes such as ‘fragmentising the story,’ i.e., only fragments of a patient’s history being available to the clinician due to time constraints, and ‘expanding one’s role,’ i.e., the clinician taking on roles beyond their traditional job roles, such as that of a social worker. While not specifically based on refugees or mental health, this model provides an interesting conceptual framework for our findings.

Previous literature investigating the barriers faced by GPs in consultations with refugees with mental health problems disregard aspects of the consultation that GPs considered to be successful. Some factors that have previously been suggested to be instrumental in the recovery of patients with refugee and other immigrant backgrounds, for example, include a good relationship between clinician and patient (Kokanovic et al., 2010; Mirdal et al., 2012; Mollah et al., 2018), patients having received some psychoeducation, the patient having a stable living situation, and effective transdisciplinary interventions and coordination (Mirdal et al., 2012; Mollah et al., 2018). Focussing on what GPs present as already contributing to successful consultations could pave the way for the development of more feasible interventions that draw on the systemic structures as well as clinicians’ strengths that are already available. Unfortunately, studies that have explored GPs’ experiences of refugees suffering from mental health problems did so prior to the main influx of refugees to Europe (Begg and Gill, 2005; Jensen et al., 2013), which, according to the European Parliament (2019) peaked in 2015, or have not focussed specifically on patients with a refugee background (Furler et al., 2010; Priebe et al., 2011; Terraza-Nunez et al., 2011; Hjørleifsson et al., 2018; Mollah et al., 2018), mental health (Johnson et al., 2008; McKeary and Newbold, 2010; Robertshaw et al., 2017), or the experiences of GPs (Wylie et al., 2018). Our study sets out to fill this gap.

The findings from this study will highlight important areas of interest that should be explored further in large scale, generalisable studies and should go toward informing interventions for GPs as well as improvements to the health care system. The risks of ineffective mental health care for refugees (Steel et al., 2011), highlight the importance of addressing this issue not only for the clinicians but also for their patient’s sake. The aim of the present study is to explore the experiences of general practitioners in Norway in clinical consultations with refugees, who present symptoms of mental health problems. The research question is: what were the main challenges and facilitators GPs perceived in clinical consultations with this patient group? We addressed this question through in-depth semi-structured interviews using a hermeneutic phenomenological approach (Langdridge, 2007; Finlay, 2011; Gadamer, 2013; Brinkmann and Kvale, 2014).

## Materials and Methods

We chose a hermeneutic phenomenological qualitative method that retains the thematic content of the interviews, to explore the GPs’ first-person perspectives, and to be able to trace subtle nuances of their perceptions (Finlay, 2011; Gadamer, 2013). This approach generates descriptive knowledge and analytic concepts from everyday experience through dialogic engagement. Each participant told their own individual story, which may not necessarily speak for all GPs or reflect the patients’ own experiences. By comparing the individual accounts, we wanted to identify both patterns of commonalities and differences in how GPs experienced clinical consultations with refugee patients, and formulate these as themes. The specific phenomenological element of our approach lies in the attitude toward, preparation for and presence in the interviews, and the use of imagination in the reading of the interviews, meaning the researcher attempts to see the experience from different perspectives to better understand it (Langdridge, 2007). The phenomenological approach does not try to explain but rather describe experiences (Laverty, 2003). The hermeneutical element in our approach implies that interpretation is necessary when we try to understand and point out the meaning of an utterance, and that the description of the participant’s lived experiences needs to be understood within the participant’s but also the researchers context (Finlay, 2011). The researcher then, in interpreting the participants’ experiences, invariably draws on their own preconceptions and understandings of the world and can never be impartial (Finlay, 2011). Since the researcher becomes the lens through which the data are interpreted, we reflect on possible preconceptions and experiences that could shape and influence how we understand the participants’ narratives (van Manen, 2014).

### Procedures

A reference group of people in relevant positions such as GPs, people with immigrant backgrounds, the Red Cross, the Bergen municipality, and psychologists gave feedback regarding the relevance of our research and helped with participant recruitment. We recruited participants through a combination of purposive and snow-ball sampling through the research group’s network as well as from obligatory GP training courses in Norway.

Throughout data collection, the first author kept a reflective diary recording experiences, reactions, and awareness of assumptions or biases following Morrow’s (2005) recommendations.

### Participants

Participants were required to have worked in a general practitioner role in Norway (either full-time or as a substitute) with at least one experience treating someone with a refugee background who presented with symptoms of mental health problems. We included one accident and emergency doctor due to the participant’s relevant experience with this patient group. Based on the authors’ previous experience with qualitative interviews, as well as previous literature (Begg and Gill, 2005; Jensen et al., 2013; Mollah et al., 2018) an approximate sample of 15 participants was estimated ahead of time. During data collection, authors reconvened and agreed that given the depth of the interviews, a sample size of 15 would be sufficient to gather a varied and relevant depiction of participants’ experiences. We approached participants via email or in person. GPs received consent forms after they had shown interest in taking part in the study, which outlined details of the study and their rights as participants. We aimed for diverse enough demographic characteristics to gain insight into a variety of experiences. Fifteen participants agreed to take part (7 female), who were working in both urban and rural areas around the country. The participants’ ages ranged from 29 to 67 (*M* = 41.7 years, *SD* = 11.1). According to self-report, nine participants were born in Norway, three in Russia, one in Denmark, one in Iraq, and one in Kurdistan. The amount of work experience ranged from 2 to 39 years (*M* = 13.6 years, *SD* = 12.1). We offered participants to hold the interview in either Norwegian or English depending on their preference. Three participants chose to be interviewed in English. Participants were remunerated 500 NOK (Norwegian krone) for their time.

### Interview Protocol

The first author conducted two pilot interviews to examine the relevance of the interview guide. Based on the pilot interviews some questions were reformulated, and we ensured that participants were familiar with the terms ‘asylum-seeker,’ ‘refugee,’ and ‘economic migrant’ before beginning the interview. Interviews were audio recorded. Interviews were semi-structured and followed an interview guide to ensure that they covered relevant and similar topics. The first author transcribed all interviews verbatim, before finishing data collection. This lead to a further revisiting and updating of the interview-guide to include new topics that appeared important throughout interviews, for example the extent to which education and training had prepared participants for working with refugee patients suffering from mental health problems. Transcriptions included information on who was speaking (researcher or participant), significant pauses, laughing or crying, and interruptions such as phones ringing. The interview began by collecting general background information about the participant (sex, country of birth, years of work experience in general medicine, amount and types of patient groups seen at the practice). This section was followed by an open question encouraging the participant to recount a consultation with a refugee patient, who displayed symptoms of mental illness. Possible follow-up questions aimed to explore the GP’s first impression of the patient, diagnoses and treatment options considered by the GP, what happened next, how the GP felt during and following the consultation, the GP’s perception of the relationship to the patient, as well as possible sources of support for the GP. The follow-up questions were not asked at every interview in the same way, as the interviewer adapted the interview allowing the conversation to follow the GPs’ narratives coherently. Following in-depth exploration of this first case, the interviewer aimed to cover the following topics if not already addressed spontaneously by the participant and if they were relevant: to what extent education and training had prepared the GP for this type of consultation, experiences providing care for refugees with trauma, and experiences using an interpreter. The interviews took approximately 60 min each.

### Researchers

This study was conducted as part of the Clinical Encounters with Refugees Suffering from Mental Health Problems research project within the Society and Workplace Diversity Group at the University of Bergen. The first author is a research fellow at the University of Bergen with a master’s degree in clinical mental health sciences from University College London and some clinical experience within mental health. Her cultural background is German and English. PB is a clinical psychologist as well as a professor of clinical psychology with many years of experience in qualitative methods. GS is a clinical psychologist and professor at the University of Bergen, as well as the project leader of the research group. She has many years of experience within the research field of migration and mental health. Both PB and GS are native Norwegians.

### Ethics Statement

This study was approved by the Norwegian Centre for Research Data (NSD Notification form: 602214). All participants gave written consent in accordance with the Declaration of Helsinki (World Medical Association, 2013). Audio recordings of interviews were stored on the secure desktop solution ‘SAFE’ (Secure Access to Research Data and E-infrastructure) (University of Bergen IT Desk, 2019), in line with the Norwegian code of conduct for information security in the health care sector. Participants received consent forms ahead of time and were encouraged to ask questions at any point.

### Analysis

Interviews were analysed in line with Braun and Clarke’s thematic analysis approach (Braun and Clarke, 2006; Clarke and Braun, 2018), following their six step guide to conducting thematic analysis including: familiarising self with the data, generating initial codes, searching for themes, reviewing themes, defining and naming themes, and producing the report (Braun and Clarke, 2006). The first author transcribed all interviews verbatim, which was considered the first stage of familiarisation with the data. Transcriptions were summarised and initial codes were noted. These were condensed according to potential overarching themes, which were reviewed by returning to the transcripts and summaries to ensure they captured the most relevant subjects discussed during the interviews. All authors turned back to the overall text to check whether voices and points of view should be added, conceptions and descriptions of themes could be developed further, or correctives to the preliminary line of interpretation represented. The authors who did not conduct the interviews, had a leading role in critically auditing the identification of thematic units. The themes were formulated and agreed upon by all authors. Finally, themes were rewritten and reported. The analysis was conducted using QSR International’s NVivo 12 qualitative data analysis software (NVivo qualitative data analysis software, 2018).

## Results

**FIGURE 2:**
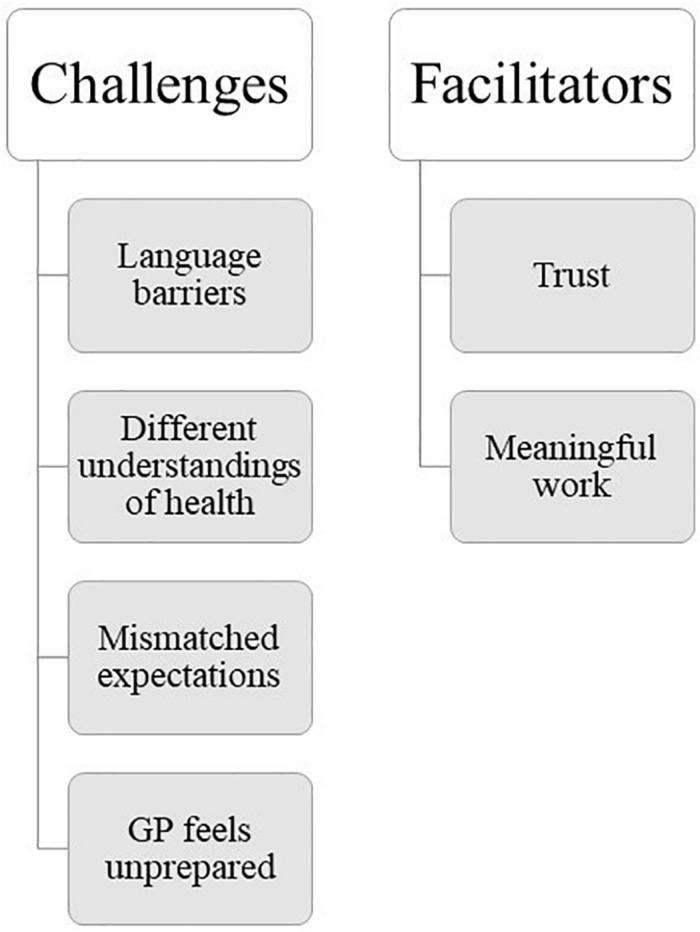
Organisation of themes.

All GPs had relevant experiences with patients with a refugee background, who, according to participants themselves, displayed symptoms of mental health problems. We present six main themes based on GPs narratives, presented under the main headings ‘challenges’ and ‘facilitators,’ to indicate the GPs’ own perceptions of whether this theme stood in the way of, or facilitated, the consultation (Figure 2). Note that initials are pseudonyms and do not have any relation to the participants’ real names.

### Challenges

#### Language Barriers Limit Our Ability to Give and Receive Help

This theme describes challenges surrounding language barriers and includes working with interpreters, which was often presented as time-consuming and difficult. Participants described communicating with their patients either in broken Norwegian or via an interpreter. Even when patients felt comfortable communicating in Norwegian a high level of language mastery was vital in order to discuss complex issues such as mental health adequately, which was often not possible. The language barrier was presented as an obstacle to diagnosing, communicating treatment options, as well as building up a trusting relationship with the patient. One participant said:

PC: So we have problem number one, which is language, right. If you meet people who are having a very difficult time then having a shared language and a common platform is such a basic thing that makes it easier for both patient and clinician.

On the flipside, speaking the same language, as well as being familiar with the patient’s culture, was considered conducive to the consultation:

PK: I mean, in this case I knew the culture, let’s say, the language, I knew everything. It made the job probably a million times easier. Both for me as a doctor but also for the patient and that also will affect the outcome.

Speaking the same language allowed this GP to pick up on ‘clues’ during the consultation, which give indications of the patient’s mental health. GPs pointed out that interpreters are rarely medically qualified making it difficult for them to present medical symptoms in a coherent way, which in turn influences the consultation outcome. Furthermore, participants pointed out that the quality of interpreters varies, and two participants described occasions where the patient spoke better Norwegian than the interpreter. One participant describes some of the challenges of working with interpreters:

PG: It depends on the quality of the interpreter. I have (pause) got the impression that the interpreter, the interpreters that we use in [our] municipality are not (pause) the same quality as those [this aforementioned] hospital uses. Many have taken only a Norwegian course, or Norwegian exam 2 or 3. I do not remember what they have taken, but it is rare that they have taken Bergen test for example or Level 3 at university so you notice a big difference. And then at the same time (pause) if the patient is sceptical of talking about these symptoms (pause) in front of an interpreter who has the same background as the patient, then it is not easy to identify these symptoms. I have a patient who has mental health problems, speaks poor Norwegian but a little, and he refuses to have an interpreter.

This participant highlights a number of challenges. First, the interpreter may not always be able to provide the necessary support leaving GP and patient at square one. Second, participants may be sceptical of working with interpreters with the same cultural background as them, possibly due to the stigma associated with certain conditions and experiences within their common culture. It is important to add, however, that many participants also praised interpreters for the good work they do and recognised their importance in cross-cultural consultations.

It was also mentioned that diagnostic tests commonly used in primary care, such as the Montgomery-Åsberg depression rating scale (MADRS) (Montgomery and Åsberg, 1979), are aimed toward people with fluent Norwegian language skills and a western understanding of health. In many cases, GPs were limited to conducting diagnostic tests via interpreters. GPs further suggested that it may be difficult to conduct effective psychotherapy through an interpreter, and that refugee patients may consequently receive a poorer quality of mental health care. A few participants were of the opinion that the Norwegian healthcare system itself is not suited to refugees with mental health problems, who lack the resources required for navigating the system, including adequate language skills.

#### When Worldviews Clash

This theme includes GPs’ impressions that patients have different understandings of the body, what constitutes health or illness, and the causes of mental illness. Some patients seemed to believe that mental illness was caused by the supernatural, such as possession by spirits or ‘jinn.’ GPs, all of which were influenced by western medicine, did not have the same beliefs. The following participant describes the difficulties of dealing with these clashing conceptualisations:

PI: And for people from low-income countries, who have little or no education, it’s more like we have such different horizons of understanding. We have such (pause) it’s so terribly difficult to communicate over a like (pause) regardless of how carefully we explain they sort of often interpret signals from the body differently.

This GP describes their experiences with some refugees, who may have low or no education. They mention ‘different horizons of understanding’ indicating that even when patient and clinician may be able to communicate verbally the meaning of their statements may be lost as a result of their different horizons of understanding health, illness, and the body. An additional challenge here was that some GPs felt like patients often did not see mental illness as a main problem in the first place:

PN: Because he did not see mental illness as a challenge, because the challenge was to deliver the children to school. The challenge was this bus that departs early. The challenge was that he could not pick up the children when he came back from school and there was no bus. Right? That was a challenge, right? The challenge was that he had no job. So, it is clear then that it becomes very difficult to explain: ‘no, you have mental health problems that you have to solve first,’ right?

All GPs acknowledged mental illness as a condition to be taken seriously, and as something that could be treated. Some of their patients, however, either did not want to discuss mental health, did not see it as a problem, or did not expect the GP to be involved in matters regarding their mental health. One of the GPs with a Middle Eastern background pointed out how culture may influence the way in which patients approach mental health. This GP explained that there are very few psychiatrists in the Middle East, and that mental illness is a taboo topic, leading to fewer diagnoses. The participant added that *‘depression is considered a usual (pause) or usual symptoms, not an illness. And then it is not easy to go a psychologist or to speak about mental health problems.’*

For some patients with a refugee background, mental health problems seemed, according to GPs, to be overshadowed by physical health problems. GPs recognised links between physical symptoms and traumatic experiences, stress, or mental illness, which they felt their patients often did not:

PL: (pause) I do not think he, well I tried to explain the connection that sometimes if one is exposed to [traumatic experiences] then it can worsen pain, for example. And he, I think he kind of went along with it a bit but I didn’t quite get the feeling that he accepted that explanation. He was very focussed on that there must be something in that foot.

#### Great Expectations and Not Living up to Them

Participants suggested that refugees’ expectations regarding the Norwegian health-care system, including the role of the GP, as well as possible treatment options and outcomes might not align with their own. Many participants felt like their patients expected of them to solve all their problems, preferably through the use of tablets or shots:

PL: And (pause) quite like (pause) yes, he most likely thinks that with pretty great certainty he says himself that it is, it must be, yes clearly something is wrong right? And then ‘you have to give me a medicine,’ that’s often the demand I get, ‘you have to take a picture or do something.’ So (pause) then, yes. So there have been a lot of challenges because to reach a dialogue which makes him receptive to talking about what he has experienced. So what I have spent a lot of time on: building the relationship and building trust with him.

The GP in this case, seemed to feel as though it would be therapeutic for the patient to discuss what they had experienced, while the patient was convinced that his problem was purely physical, which ought to be solved through medication and medical tests. Other participants described similar discrepancies in expectations. Consequently, some were required to spend time during consultations explaining the Norwegian healthcare system to their patients and clarifying what a GP is capable of helping with. One participant described the effect the mismatched expectations had on them:

PM: Yeah, the effect on me (pause) well, I (pause) I felt a bit (pause) I wouldn’t say helpless, but I felt it was, I felt that it was very difficult to help him, because we didn’t even have a shared understanding of what that help could be.

A few patients made GP appointments in order to discuss administrative issues such as letters they had received from the Norwegian Labour and Welfare Administration (NAV) or electricity bills. Many of the GPs expressed going beyond their traditional roles as GPs to provide adequate care for their refugee patients. However, one GP also warns of the consequences of being willing to go above and beyond for this patient group:

PH: So we took someone in privately to work in our garden and maybe invited him for dinner. And that was fine. But it can become very, it is very exhausting, because then you are both a doctor and you get to know, you also develop a private relationship, so there we sort of (pause) had a balancing act. We have (pause) my wife has taken, supported a few refugees here [in this place] among others a single mother with a small child who is adjusting very well. But you must, you must set boundaries there, you cannot become too involved.

#### I Was Not Prepared for It

Most participants agreed that their university education had not adequately prepared them for working with refugees suffering from mental health problems. Some GPs leaned on the information in the patient’s medical files in order to make a more confident clinical decision, but files were often not available or non-existent. Similarly, GPs mentioned that in the case of a Norwegian patient they might involve the patient’s social network to gather more information, which in the case of refugees was often difficult, either because they had no network or they were difficult to contact. One participant describes the first meeting with a refugee patient, who suffered from mental health problems:

PJ: I was not prepared for it. So, it was a big shock. I felt like I almost couldn’t use anything of what I had learned during my education and that was very strange, because I had some expectations that during my placement and many other situations I would use the knowledge that I had, but here I felt as though I had very little knowledge.

Some participants reported feeling uncertain about their clinical decisions. In some cases this was related to the lack of information, which lead to a fear of making the wrong decision:

PA: It was well, what was difficult was that I felt sort of that I was making decisions based on lacking information. That was it. I was afraid of not doing the right thing in a way.

This participant later added:

PA: That is our world. We do things every day that we can’t do. We just need to try to manage, right?

The participant seemed to feel like he was missing vital information in order to make a confident clinical decision, while also recognising this as a part of the job of a GP, to do things they are not prepared for. Some participants suggested that the education system could have done better in preparing them for the challenges related to working with this patient group, while a few felt like there was not much else the education system could have done to prepare them. In fact, many reported that while their education may not have specifically prepared them for these cases, their experiences had:

*PL: I am educated in [another country]. So in that sense it is, I have some experience with non-Norwegian culture and that is living in another culture [*…*]. It demands a lot extra from you as a clinician to manage that in a good way, that’s something you just have to learn in practice I think. That is how it is with a lot of things. I probably wasn’t prepared for it when I finished my studies. What it was like to work with an interpreter, and work with, work with, yes very different values or other, in a way, thoughts about how the system should work and those with a different culture it is like, yes, you sort of learn as you go along.*

However, GPs experiences varied, and none of the participants outlined having received specific instructions or guidelines on how to work with a refugee patient suffering from mental health problems, including how to effectively work with an interpreter.

### Facilitators

#### Trust as a Bridge

Many of the challenges that GPs discussed seemed to be improved when patient and GP had established a trusting relationship. In the following case, a trusting relationship led to better communication:

PI: Yes, she, after a while we developed a trusting relationship and in a way she was able to speak to me about what had happened.

The most important factor in establishing a trusting relationship, however, was time. Most participants pointed out that building up a relationship often took a long time, required continuity of care, and taking the patient seriously. One participant described the process of building up such a relationship:

PE: Many patients feel that they do not have enough time to (pause) that it is in a way (pause) that it is a challenge with a GP because many GPs set aside 10 min or 15 min or 20 min. I think when I realise that I have such a patient (pause) something to do with such a patient, then at the first meeting I say that ‘we’ll set up a new appointment very soon, and we’ll arrange a double appointment and then we’ll become more familiar with one another and then we’ll establish your needs’ and (pause) I feel that in a way saying that I will try to help you within these frameworks here is very important. Importantly, I have at least experienced that it is a very important thing for them to hear, that they feel safe and seen and that even if we do not get to do so much, that they leave the consultation and feel that there is someone who has tried to help them at least, I think that is of great value.

While most GPs recognised the importance of a good alliance between clinician and any patient, they also acknowledge that this was harder to establish than with Norwegian patients:

PL: But it is probably also easier to do because of, it is easier to establish a relationship [with Norwegians] because of language and, there is no language barrier there. So (pause) then one can assume that it also contributes to having a (pause) it is easier to establish a trusting relationship then. So, if you imagine that you are trying to convey the message that, for example regarding the treatment of back pain, or treatment of pain through exercise or, or light activity, no tablets no pills, no such thing, and then to convey that message with an importance that the patient believes in then it is important to have a trusting relationship. And building that relationship is maybe a little easier when you do not have, when you are just one on one, do not have an interpreter in the room, where you always have an open dialogue, where you understand each other well. If you have an interpreter in the room it will probably be a little more difficult to build that trust in order to be able to convey such a message to the patient so that the patient accepts it.

This GP suggested that a trusting relationship between GP and patient seems to encourage the patient to adhere to treatment. This participant also discusses that an interpreter can stand in the way of establishing such a relationship, while another participant stated how interpreters could support the consultation. Overall, many participants pointed out that establishing a trusting relationship with a patient with a refugee background was possible and very valuable.

#### These Consultations Are Deeply Meaningful

While acknowledging the challenges associated with this type of work, most GPs felt like working with refugee patients was a meaningful and interesting part of their job, and participants highlighted that the desire to help their patients was not outweighed by the previously mentioned challenges. One GP recognised this type of work as a privilege:

PJ: I want a job where I get goose bumps, if I feel like ‘oh this has touched me a lot.’ Yes, because then I feel most alive and I need such a job, you see? So I think it makes a lot of sense to be given the privilege of hearing those stories, because I think to be allowed to witness something that is so personal I think is very, very meaningful and rewarding. It’s a gift.

Within the same sentence, another GP presented the work as both ‘hard work’ as well as the reminder of why they became physicians:

PM: I’ve mostly come away from these meetings with a feeling that I actually did something worth doing today. I, you know, I was in the right spot. I was supposed to be there and I could probably do it better, but it was the right place for me to be, and it was a good day. So, that, I think that would be important for me to add, because otherwise the story is often presented as, you know, that’s just hard work for physicians, but it is also a reminder why we became physicians, I think.

These and similar comments show that the GPs in these interviews, while recognising certain challenges, were often willing to go above and beyond in their job to support this patient group:

PE: But one becomes willing to go above and beyond for those, yes, in this practice here we are perhaps more preoccupied given where we work in [this place] and what we, in a way, want to represent, so we are interested in going above and beyond for those patient groups that need us.

## Discussion

### Summary of Themes

This study explored GPs’ perceived challenges and facilitators in clinical consultations with refugees suffering from mental health problems through individual semi-structured interviews. Resulting themes are presented under the overarching headings ‘challenges’ and ‘facilitators,’ to indicate participants’ own opinions of whether the issue was a factor that helped or stood in the way of effective consultations.

Challenges included themes related to language barriers, different understandings of health and illness, mismatched expectations, and feelings of being unprepared. Facilitators included themes related to developing a trusting relationship and finding the work meaningful and interesting. Themes were interrelated and impacted on one another. For example, language barriers seemed to complicate other aspects of the consultation, for example clarifying what constitutes health and illness as well as the patient’s expectations of health-care. Language barriers, different understandings of illness, and mismatched expectations of health-care seemed to make working with refugee patients more challenging than with Norwegian patients and contributed to GPs’ feelings of being unprepared. Similarly, GPs discussed that it was easier to build trust, an important element of a successful consultation, with Norwegian patients often due to having a shared language. However, while time-consuming to establish, a trusting relationship between clinician and refugee patient facilitated communication. Finally, many GPs found consultations with refugees meaningful and interesting, partly due to exactly those challenges presented above, which made them feel like they could make a valuable difference in their patients’ lives.

Language barriers posed an important challenge both in our study and in previous literature (Jensen et al., 2013; Wylie et al., 2018). The ‘circling the undefined’ model highlights that information needs to be shared in order for clinician and patient to clarify those issues that are fundamental to the consultation, such as what constitutes health and illness (Rothlind et al., 2018). Their theme ‘fragmentising the story’ suggests that a large obstacle in sharing information is a lack of time. While language is not specifically referred to in Rothlind et al’s model, our study suggests that language barriers may also pose a significant obstacle in the process of sharing information. Working with interpreters is meant to support the sharing of information between clinician and patient, however, our participants suggested that working with interpreters had both advantages and disadvantages. Previous literature suggests similar. When interpreters are introduced into the consultations they create a triadic therapeutic alliance, which when successful may enhance the consultation, but when unsuccessful may stand in the way of a healthy therapeutic alliance between clinician and patient (Miller et al., 2005). Miller et al. (2005) suggest that interpreters with a refugee background, who have been appropriately trained for working in a clinical setting should be employed and can be a great asset to intercultural consultations. However, language barriers seemed to go beyond verbal communication and affected other diagnostic procedures. According to our participants, diagnostic tools used in primary care in Norway are rarely culturally validated or even translated into other languages. Participants reported using standard assessment tools, such as the MADRS (Montgomery and Åsberg, 1979) translated through an interpreter during the consultation. This procedure is not considered a reliable, valid, and acceptable way of translating diagnostic questionnaires (Hambleton and de Jong, 2003), and could arguably lead to misdiagnoses. This highlights the importance of employing appropriately trained interpreters, as well as making culturally validated diagnostic tools readily accessible in primary care.

Some GPs in both our and previous studies (Jensen et al., 2013), found that their understandings of the causes of mental illness differed from those of their patients, who sometimes believed in possession of evil spirits, or ‘jinn.’ Furthermore, they reported that some of their patients did not speak openly about mental illness and often presented symptoms in terms of physical problems, known as ‘somatisation’ (Kirmayer, 2001), which complicated diagnostic decision making. It has previously been reported that ways of explaining and presenting mental illness seem to differ between cultures and languages. In Arabic, for example, it may be more common to describe sadness in terms of having a ‘blind’ or ‘squeezed’ heart, while in Kurdish, low mood may be portrayed in terms of being short of breath or the world becoming dark (Hassan et al., 2015). The observation that refugees somatise their distress is not a new insight (Fuller, 1993; Kokanovic et al., 2010; Farley et al., 2014). However, it has been suggested that it may be a generalisation made by people in Western cultures (Kirmayer, 2001). Kirmayer (2001) claims that ‘if there is any validity to this generalisation, it can only be because Westerners (who themselves comprise extremely diverse and divergent cultural groups) share some distinctive values or practices that contribute to the obverse of somatisation, which has been termed psychologisation’ (p. 23). This idea is also to some extent echoed in Rothlind et al. (2018) theme ‘culture blaming and explaining,’ which suggests that clinicians and patients in intercultural consultations may have the tendency to (erroneously) attribute each other’s behaviours to culture, which prevents them from exploring each other’s perspectives and motivations more thoroughly. While not openly stated by any of the participants in our study, it is possible that they, as well as we, also have the tendency to engage in ‘culture blaming and explaining.’

Our participants experienced that their patients often had high expectations of them, of being cured quickly, and of receiving pharmacological treatment. Especially the expectation of receiving pharmacological treatment has been mentioned in previous literature (Jensen et al., 2013). When left unaddressed, this mismatch of expectations may lead to dissatisfaction and low compliance with treatment. While not in the context of refugees and mental health, Ali et al. (2006) study provides an interesting insight into the effects of mismatched expectations in intercultural consultations. They suggest that patients living in the United Kingdom with a different cultural background, who were fluent in English and familiar with the United Kingdom health-care system, compared to those who were not, were more satisfied with their treatment. The authors explain this in terms of expectations claiming that patients who are familiar with the health-care system have more realistic expectations of treatment. This is supported by Rothlind et al.’s (2018) ‘circling the undefined’ model, which suggests that part of the perceived complexity of intercultural consultations is a result of clinician and patient having a silent agreement on their expectations from treatment, which may not align. The model also describes a mismatch between what clinicians and patients expect their own roles and responsibilities to be, often leading to confusion and conflict.

Most GPs in our study stated that they felt their education had not adequately prepared them for working with refugees suffering from mental health problems. Other studies found that clinicians lack a uniform procedure to deal with such consultations (Wylie et al., 2018), lacked support or resources (Begg and Gill, 2005), and sufficient education and supervision (Fuller, 1993). While participants in our study pointed out that their education may not have prepared them for this work theoretically, they believed that their practical experience had. Some participants also argued that education alone could not prepare them. Nevertheless, the majority of participants agreed that preparation with regards to, for example, the proper use of interpreters would have been helpful. Similarly, the ‘circling the undefined’ model outlines that roles and responsibilities become blurred, and the clinician may find themselves taking on roles their education had not prepared them for, such as a social worker, psychologist, and/or language teacher, among other things, which could lead to stress and less job-satisfaction (Rothlind et al., 2018). Importantly, it has been shown that the hindering effect of the lack of knowledge was decreased after taking cross-cultural mental health training (Bäärnhielm et al., 2014), highlighting the importance of such training in improving GPs confidence and competence in working with this patient group.

Alongside the challenges presented above, participants also reported factors that they felt contributed to, what they considered, successful clinical consultations with refugees suffering from mental health problems. Participants suggested that a trusting relationship encouraged patients to share more personal experiences, which helped the GP gather a clearer idea of the patient’s experiences and symptoms and identify appropriate treatment options. Trust was presented in one study on refugee-background young people accessing mental health services as both a challenge and a facilitator (Colucci et al., 2015) and has also been mentioned in similar studies with immigrant patients in a general health context (Priebe et al., 2011; Mirdal et al., 2012; Robertshaw et al., 2017). While our participants discussed trust in a mostly facilitating context, these studies suggest that while the presence of trust can be invaluable, the absence of trust can be detrimental. Trust is, of course, not an element that is unique to the relationship between GP and patient with a refugee background. However, the importance of trust may be even more conducive and the lack of trust may be even more detrimental in a cross-cultural consultation. Furthermore, our participants pointed out that establishing a trusting relationship took time, something GPs often lacked due to time constraints on consultations.

Most of our participants found it important to highlight that they thought working with refugees with mental health problems is meaningful and interesting. This was partly due to exactly those challenges presented above, which made GPs feel like they were able to make a valuable difference in patients’ lives. Similarly, primary health care workers and administrative staff at a specialist refugee health care service in Australia reported that despite challenges associated with their work, they were committed and enthusiastic about working with refugees (Farley et al., 2014). As far as we are aware, this topic has not been addressed in previous research exploring GPs experiences of providing care to refugees with mental health problems in a non-specialist health care setting, and is therefore an important contribution to the literature. It is important to remember, however, that while GPs in the current study find their work meaningful, they are nevertheless limited by both the challenges mentioned above as well as challenges on a systemic level that need to be addressed. Schwartz and Sharpe (2006) point out that the health-care system does not foster the quality of ‘practical wisdom,’ which is the ability to know what to do as well as wanting to do it because it is in line with one’s values as opposed to just aiming to meet targets or being motivated by financial incentives. They suggest that doctors who achieve practical wisdom, who see their work as both meaningful and interesting, and whose work provides them with the opportunities to act in line with their own values, may consequently experience higher work satisfaction and may be able to provide higher quality care to their patients.

### Reflexivity and Limitations

Working with ambiguous texts, such as these interviews, is a process that is vulnerable to observer bias and researchers may ‘see what they expect or (subconsciously) want to see’ in the data (Binder et al., 2016, p. 4). To address this in a hermeneutic phenomenological approach the researcher engages in a self-reflective dialogue regarding their own preconceptions and how these influence the research procedure (Laverty, 2003; Finlay, 2011; Gadamer, 2013). Following Morrow’s (2005) recommendations, the first author kept a reflective diary recording experiences, reactions, and awareness of assumptions or biases. Some relevant reflections are presented below.

All authors had western backgrounds, which were similar to the majority of the participants. It is reasonable to assume, therefore, that they shared a relatively similar understanding of illness based on western medicine. These similarities may have facilitated the examination, but this assumption may also have led to over reliance on these perceived similarities when interpreting participants’ statements (Merriam et al., 2001). Furthermore, none of the researchers were GPs themselves: PB and GS are experienced psychologists, and SH has worked with people with mild to moderate mental health problems. However, the authors’ experiences of working with clients in a clinical setting may have allowed them to judge the most relevant themes to cover in the interviews with GPs, while their differences may have allowed them to interpret GPs’ narratives through a different lens than someone within general medicine. Not sharing characteristics, role, or experience with participants can make the author an ‘outsider’ (Dwyer and Buckle, 2009). Being an ‘insider’ is said to ensure the trust and acceptance of the participants, allowing one to reach deeper and more meaningful narratives, but being an ‘outsider’ has its own advantages. An ‘outsider,’ for example, is not influenced by their own experiences and is therefore more open-minded to being guided by the participants’ own experiences (Dwyer and Buckle, 2009).

In a discussion about dominance, Kvale describes the qualitative interviewer as using emotional rapport as a ‘Trojan horse’ to get behind defence walls of the interview subjects, who in turn may share information they later regret sharing (Kvale, 2006). The researcher holds power by setting the terms of the interview, deciding which questions to ask, and determining to some extent the direction of the interview. GPs may be used to being in a position of power in relation to their patients, but their role may not be as clear when sitting opposite a researcher. The researcher’s influence on the participants and power over the interview was an important realisation, which led to actively informing the participants to only share as much as they felt comfortable with.

The study has some limitations that should be taken into consideration when interpreting the results. First, participants were given the choice of speaking Norwegian or English during their interviews. The majority of participants chose to hold the interviews in Norwegian. Norwegian is not the interviewer’s (SH) first language and may consequently have led to linguistic misunderstandings. Therefore, SH was shadowed by an experienced qualitative researcher, who speaks fluent Norwegian, during the first three interviews to ensure the quality of the interview procedure and evaluate the language barrier between interviewer and participants. SH was able to communicate easily and was understood well by participants. Obvious misunderstandings were addressed during the interviews, and any linguistic misunderstandings arising after interviews were clarified with the help of the Norwegian speaking co-authors and research assistant during the transcription and analysis phase. The advantage of allowing the participants to share their experiences in the language they are most comfortable with outweighs, in our opinion, the disadvantage of Norwegian not being the interviewer’s first language.

Secondly, since participation in the study was voluntary we may have recruited mostly participants who were sympathetic to the topic of our research. Our theme related to finding the work meaningful and interesting may be a consequence of this. Furthermore, given the socio-political sensitivity of the topic of immigration and mental health, social desirability may have influenced the participants’ responses. However, we successfully recruited a group of otherwise diverse participants, in terms of age, experience, gender, and ethnicity, which allowed the collection of a variety of narratives.

Finally, the interview guide, data collection, and analysis had an unavoidable subjective dimension, and were therefore influenced by the experiential horizon of the authors. Given the hermeneutic phenomenological approach of this study, however, this is not necessarily a disadvantage. The author is the lens through which the data are interpreted (Laverty, 2003), and through reflexivity we aimed to expand and transform as much as possible of the subjective dimension into an opportunity for exploration.

### Conclusion and Future Avenues

According to our participants, the main challenges they experienced in consultations with refugees suffering from mental health problems related to language barriers, having different understandings of illness, having different expectations of health care, and GPs feeling unprepared for this work. A trusting relationship between patient and GP was said to facilitate consultations, and many GPs found working with refugees with mental health problems meaningful and interesting. Considering both facilitating factors alongside challenges in futures studies may lead to a more balanced view on how to develop interventions to improve mental health care for refugees, which focus not only on what needs to be improved but also draws on clinicians’ strengths and encourages those aspects of consultations that are already contributing to successful outcomes. Furthermore, it is vital that we explore systematically what effect patients’ backgrounds have on diagnoses and treatment options offered by clinicians, including the validity of frequently used diagnostic tools. These findings should be integrated with patients’ own experiences to inform and tailor interventions accordingly. As a result of globalisation, in the wake of war, and more recently even climate change, migration is becoming a steadily increasing phenomenon. The importance of understanding how we can better address the challenges associated with providing health-care for immigrants, including refugees, must quickly become a priority in medical educational curricula. The way we choose to address these issues will hold important implications for public health and the well-being of both clinicians and people with a refugee background seeking help for mental illness.

## Data Availability Statement

The datasets generated for this study will not be made publicly available because there is confidential information in the data. Requests to access the datasets should be directed to the corresponding author.

## Ethics Statement

The study was reviewed and approved by the Norwegian Centre for Research Data (NSD). The participants provided their written informed consent to participate in this study.

## Author Contributions

SH, P-EB, and GS contributed to the conception and design of the study, conducted the analysis, and edited the manuscript. SH conducted all interviews. SH wrote the first draft of the manuscript. All authors contributed to manuscript revision, read and approved the submitted version.

## Conflict of Interest

The authors declare that the research was conducted in the absence of any commercial or financial relationships that could be construed as a potential conflict of interest.
